# Data on the bisphenol A adsorption from aqueous solutions on PAC and MgO~PAC crystals

**DOI:** 10.1016/j.dib.2018.10.033

**Published:** 2018-10-17

**Authors:** Bahram Kamarehie, Syed Mehrdad Seifi Tizabi, Rouhollah Heydari, Ali Jafari, Mansour Ghaderpoori, Mohammad Amin Karami, Afshin Ghaderpoury

**Affiliations:** a Department of Environmental Health Engineering, School of Health and Nutrition, Lorestan University of Medical Sciences, Khorramabad, Iran; b Razi Herbal Medicines Research Center, Lorestan University of Medical Sciences, PO Box 68149-89468, Khorramabad, Iran; c Nutritional Health Research Center, Lorestan University of Medical Sciences, Khorramabad, Iran; d Student Research Committee, Shahid Beheshti University of Medical Sciences, Tehran, Iran

**Keywords:** Bisphenol A, Adsorption, Isotherm, HPLC, Aqueous solutions

## Abstract

The compounds of endocrine disrupting are one of the important pollutants in the environment. These pollutants, even at extremely low concentrations, have significant effects on humans, animals and the environment. The main goal of this work was to study the performance activated carbon coated with MgO in the bisphenol A adsorption from aqueous solutions. The leading variables investigated were initial concentration of bisphenol A (20–100 mg/L), PAC and MgO~PAC (2–6 g), contact time (10–60 min), and pH (3–11). The residue concentration of bisphenol A was measured by temperature High-Performance Liquid Chromatography. The maximum adsorption of bisphenol A over PAC and MgO~PAC crystals was 9.2 mg/g and 22.28 mg/g, respectively. Based on BET, the surface area of PAC and MgO~PAC crystals were found to be 450.3 m^2^/g and 378.21 m^2^/g, respectively. By increasing initial concentration of bisphenol A, the adsorption decreased. The study findings showed that the Langmuir model and the pseudo-second-order model were a fit model to the experimental data, respectively.

**Specifications table**

**Table:** 

Subject area	*Environmental Chemistry*
More specific subject area	*Adsorption*
Type of data	*Table, figure*
How data was acquired	*HPLC (Shimadzu Corp., Kyoto, Japan) consisting of a quaternary pump (LC-10ATvp), UV–vis detector (SPD-M10Avp), vacuum degasser and system controller (SCL-10Avp)*
Data format	*Raw, analyzed,*
Experimental factors	*The experiments of bisphenol A adsorption were done in batch conditions. The leading variables investigated were initial concentration of bisphenol A, PAC and MgO~PAC, contact time, and pH. the residue concentration of bisphenol A was measured by temperature High-Performance Liquid Chromatography*
Experimental features	*(I) Add magnesium nitrate hexahydrate [52.5 g] to a distilled water [1 L], (II) Add sodium hydroxide [3 mL, 1 N] to the solution prepared in the previous step, (III) Mix the solution prepared to create a mixture of gelatin homogeneous from magnesium hydroxide [5 min], (IV) Add PAC [50 g] and mix it [1 h], (V) After separating the produced precipitate, it dried in an oven [100* *°C, 3 h], and (VI) To convert Mg(OH)2 to MgO, it was calcinated [500* *°C, 2 h].*
Data source location	*Khorramabad, Lorestan University of Medical Sciences, Iran*
Data accessibility	*Data are included in this article*

**Value of the data**

● The information obtained the data of this article showed that by the modification of conventional absorbents can be their ability to adsorption various pollutants from aqueous solutions.
● The acquired data of this article can be used to complete the data on the adsorption of Phenolic compounds from wastewater and industrial effluents.
● The information of the isotherms and kinetics will be informative for predicting and modeling of the Phenolic compounds adsorption from aqueous solutions.

## 1 Data

Phenolic compounds are one of the most important environmental pollutants that have low biodegradability. These compounds have harmful effects on human, animal and environmental health. One of the common ways to remove these compounds is adsorption processes. So far, various adsorbents have been used to remove phenolic compounds from aqueous solutions. Therefore, in this study, adsorbents PAC and MgO~PAC crystals were selected to remove phenolic compounds.

## 2 Experimental design, materials, and methods

### 2.1 Materials

In this study, these chemicals were used magnesium nitrate, bisphenol A (C_15_H_16_O_2_), sulfuric acid, acetic acid, power activated carbon (PAC), acetonitrile, dichloromethane, iron chloride tetrahydrate, iron chloride tetrahydrate, and alizarin red S. the all chemicals with high quality were purchased from the Sigma-Aldrich and Merck companies.

### 2.2 Preparation of MgO~PAC

Previous studies were used to modify power activated carbon (PAC) with manganese oxide (MgO) [1], [2], [3]. This method was used to modify: (I) Add magnesium nitrate hexahydrate [52.5 g] to a distilled water [1 L], (II) Add sodium hydroxide [3 mL, 1 N] to the solution prepared in the previous step, (III) Mix the solution prepared to create a mixture of gelatin homogeneous from magnesium hydroxide [5 min], (IV) Add PAC [50 g] and mix it [1 h], (V) After separating the produced precipitate, it dried in an oven [100 °C, 3 h], and (VI) To convert Mg(OH)_2_ to MgO, it was calcinated [500 °C, 2 h]. After the above steps, the power activated carbon coated MgO was stored in a bottle for later use. After coated, MgO~PAC characterizations were determined.

### 2.3 Adsorbent characterization

The metal-organic frameworks of ZIF-8 and Uio-66 were determined by XRD, SEM. BET surface area and total pore volumes of the adsorbents were determined from N_2_ adsorption isotherms at 77 K.

### 2.4 The experiments of bisphenol A adsorption

**Fig. 1:**
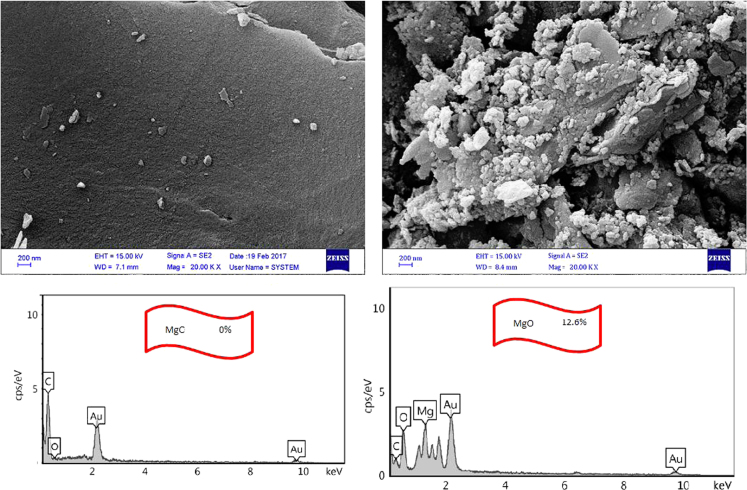
The spectra of XRD and the images of SEM of PAC and MgO~PAC crystal.

**Fig. 2:**
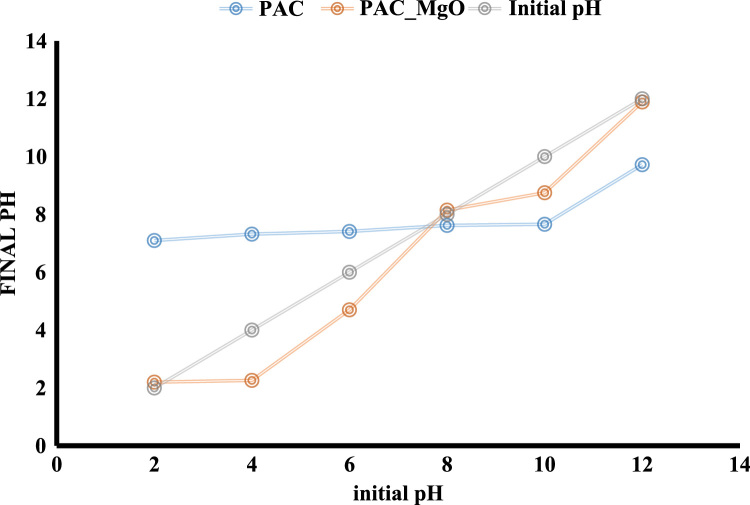
Determination of pH_zpc_ for PAC and MgO~PAC.

**Fig. 3:**
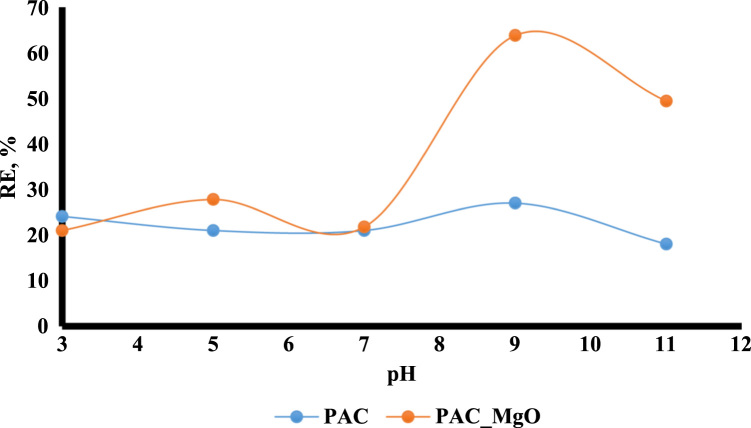
The effect of solution pH on bisphenol A adsorption with PAC and MgO~PAC crystals.

**Fig. 4:**
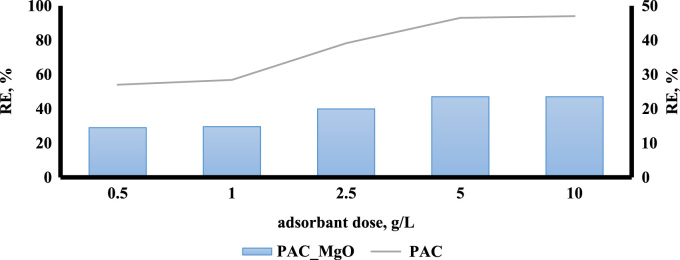
The effect of adsorbent dose of PAC and MgO~PAC crystal on bisphenol A adsorption.

**Fig. 5:**
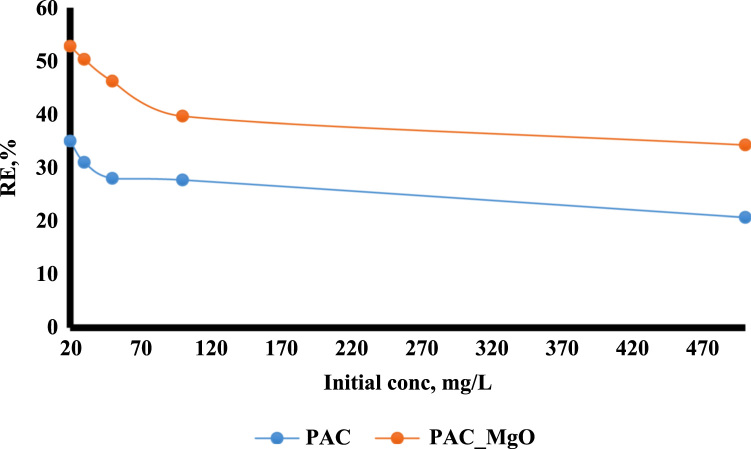
The effect of initial concentration of bisphenol A with PAC and PAC~MgO crystals.

**Fig. 6:**
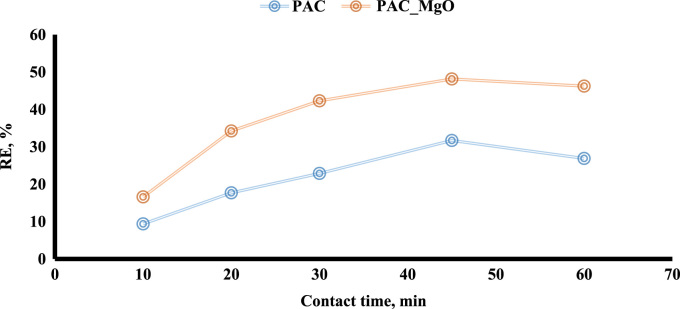
The effect of contact time on bisphenol A adsorption with PAC and PAC~MgO crystals.

The adsorption of bisphenol A by PAC and MgO~PAC was investigated. The experiments of bisphenol A adsorption were conducted in batch conditions. The leading variables investigated were initial concentration of bisphenol A (20–100 mg/L), PAC and MgO~PAC (2–6 g), contact time, and pH (3–11). At the end of the adsorption process, the residue concentration of bisphenol A was measured by temperature High-Performance Liquid Chromatography (HPLC) [1], [4], [5], [6], [7], [8], [9], [10], [11], [12], [13], [14], [15], [16], [17], [18], [19], [20], [21], [22]. Finally, Eq. (1) was used to determine the removal efficiency of bisphenol A on ZIF-8 and Uio-66:

(1) Removalefficiency,%=(C0−Ct)C0

where, *C*_0_ and *C_e_* are the initial and final bisphenol A concentrations in solution (mg/l), respectively. To determine the pH_ZPC_ of adsorbents, the steps were carried out as follows: (i) Preparation of potassium nitrate solution [50 mL, 0.01 M], (ii) Preparation of potassium nitrate solutions with a pH 2–12, (iii) Add absorbers to it [0.2 g/l], (vi) Stirring the mixture for 24 h, (v) pH measurement after this time, and finally (iv) plot initial pH Vs. final pH. The relationships shown in Table 1 were used to calculate isotherms and kinetics of bisphenol A adsorption on ZIF-8 and Uio-66. Fig. 1 shows XRD spectra and SEM image of PAC and MgO~PAC. According to BET, the specific surface area PAC and MgO~PAC were 450.3 m^2^/g and 378.21 m^2^/g, respectively. Also, total-pore volume and average-pore diameter of PAC and MgO~PAC were 0.553 cm^3^/g, 0.455 cm^3^/g and 5.1 nm, 4.7 nm, respectively. In Fig. 2, pH_zpc_ are presented for PAC and MgO~PAC. Fig. 3 shows the effect of solution pH on bisphenol A with PAC and MgO~PAC crystals. Fig. 4 presents the effect of adsorbent dose of PAC and MgO~PAC crystal on bisphenol A adsorption. Fig. 5 shows the effect of initial concentration of bisphenol A with PAC and PAC~MgO crystals. Fig. 6 presents the effect of contact time on bisphenol A with PAC and MgO~PAC crystals. Constants of kinetic models for the adsorption of bisphenol A onto PAC and MgO~PAC crystals are shown in Table 2. Constants of isotherm models for the adsorption of bisphenol A onto PAC and MgO~PAC crystals are shown in Table 3.

**Table 1:** Empirical formulas of the applied isotherm models [18].

**Isotherm models**	**Formula**	**Plot**
Langmuir	$C_{e}/q_{e}=1/K_{L}+(a_{L}\times C_{e}/K_{L})$	$\frac{C_{e}}{q_{e}}\;\;Vs.\;\;\;C_{e}$
Freundlich	$Logq_{e}=\;\log\;K_{F}+(n_{F}\times \;\log\;C_{e})$	Log *q_e_* Vs. log *C_e_*

**Table 2:** Constants of kinetic models for the adsorption of bisphenol A onto PAC and MgO~PAC crystals.

**Adsorbents**	***q**_**e**_***[mg/g]**	**Pseudo-first-order**			**Pseudo-second-order**		
		***q***_**cal**_	***K***_**1**_**[ 1/min]**	***R***^**2**^	***q***_**cal**_	***K***_**2**_**[ 1/min]**	***R***^**2**^
PAC	5.068	3.27	0.0333	0.93	5.49	0.0157	0.994
MgO~PAC	14.03	5.21	0.044	0.862	17.89	0.0123	0.987

**Table 3:** Constants of isotherm models for the adsorption of bisphenol A onto PAC and MgO~PAC crystals.

**Adsorbents**	**Langmuir**				**Freundlich**		
	***q**_**m**_***[mg/g]**	***b* [L/mg]**	***R***^**2**^	***R**_**L**_*	***K**_**F**_*	***n***	***R***^**2**^
PAC	9.2	0.124	0.927	0.074	7.05	0.66	0.849
MgO~PAC	22.28	0.268	0.991	0.036	2.43	0.62	0.989
