# Supplementary material for: Data on the bisphenol A adsorption from aqueous solutions on PAC and MgO~PAC crystals
Source: Data Brief. 2018 Oct 17;21:746–52. doi: 10.1016/j.dib.2018.10.033 (PMC6214841; doi:10.1016/j.dib.2018.10.033)
Supplement: Supplementary file 1 — Transparency document [file mmc1.docx]

Dear Professor,

Editor of **Data in Brief**

The authors of this article declare that they have no conflict of interests.

**Data on the bisphenol A adsorption from aqueous solutions on PAC and MgO~PAC crystals**

Bahram Kamarehie^1^, Syed Mehrdad Seifi Tizabi^1^, Rouhollah Heydari^2^, Ali Jafari^1^, Mansour Ghaderpoori^1, 3, *^, Mohammad Amin Karami^1^, Afshin Ghaderpoury^4^.

1. Department of Environmental Health Engineering, School of Health and Nutrition, Lorestan University of Medical Sciences, Khorramabad, Iran
2. Razi Herbal Medicines Research Center, Lorestan University of Medical Sciences, PO Box 68149-89468, Khorramabad, Iran
3. Nutritional Health Research Center, Lorestan University of Medical Sciences, Khorramabad, Iran
4. Student Research Committee, Shahid Beheshti University of Medical Sciences, Tehran, Iran
